# Intergeneric Introgression Enhances the Adaptive Potential of Nine-Spined Stickleback (Pungitius pungitius)

**DOI:** 10.32607/actanaturae.27528

**Published:** 2025

**Authors:** A. V. Nedoluzhko, F. S. Sharko, S. M. Rastorguev

**Affiliations:** European University at St. Petersburg, St. Petersburg, 191187 Russian Federation; National Research Center “Kurchatov Institute”, Moscow, 123182 Russian Federation; Pirogov Russian National Research Medical University of the Ministry of Health of the Russian Federation, Moscow, 117997 Russian Federation

**Keywords:** introgression, hybridization, nine-spined stickleback, Pungitius pungitius, adaptation, three-spined stickleback, Gasterosteus aculeatus

## Abstract

Over the past decades, number of evidences has accumulated that demonstrates the importance of genomic introgression between relatively distant eukaryote species, including the introgression of teleost fish species; the three-spined stickleback (*Gasterosteus aculeatus*) and the nine-spined stickleback (*Pungitius pungitius*). The whole-genome datasets of both teleost species give reasons for suggesting that the marine population of nine-spined stickleback increases its adaptive potential to the marine environment through introgression with the anadromous three-spined stickleback. These findings demand a reinterpreting of the mechanisms of evolution towards a process in which organisms acquire new traits not only through longterm accumulation and selection of spontaneous mutations, but also via introgression from other species and ecological forms.

## INTRODUCTION

Natural interspecific hybridization giving rise to viable and fertile offspring occurs relatively frequently, even between genetically distant plant [1, 2, 3] and animal [4, 5, 6, 7, 8] species. In most cases, it remains unclear whether this genetic exchange is a result of random processes or whether it plays a crucial role in the species’ adaptation to changing environmental conditions or to the conquering of new ecological niches [9, 10].

In some cases, it has been demonstrated that introgressive hybridization can be adaptive, leading to the emergence of morphologically and physiologically distinct forms that contain the genetic material of both parental species. Such adaptive mechanisms have been demonstrated to exist only in hybrid forms with a significant level of introgression from both parents [11, 12, 13, 14]. Meanwhile, it still remains unclear whether interspecific introgression causing no noticeable morphological changes is adaptive or occurs occasionly, with its traces in the gene pool of a species eventually fading over time.

Previously, we detected introgressive hybridization in White Sea populations of the three-spine (*Gasterosteus aculeatus*) and nine-spine (*Pungitius pungitius*) sticklebacks [15, 16]. It was suggested that this introgression contributes to the emergence of phenotypes of nine-spine stickleback adapted to salinity. This level of introgression caused no significant morphological changes and could only be identified by whole-genome sequencing. In that case, it also was unclear whether the emergence of introgressed loci in the nine-spined stickleback was the result of random hybridization or was adaptive [16].

In this study, we detected traces of introgression of the three-spined stickleback into the genotypes of Holarctic populations of the nine-spined stickleback based on previously reported genomic datasets of marine and freshwater nine-spined stickleback specimens [17] and using D-statistics analysis, also known as the ABBA-BABA test [18]. Furthermore, the observed level of introgression was much higher for marine populations of the nine-spined stickleback than it was for freshwater ones, suggesting that introgression between these two species is adaptive.

## EXPERIMENTAL

**Bioinformatics analysis**

In this study, we used whole-genome sequencing data from 870 nine-spined stickleback specimens that had been obtained by Feng et al. and deposited in the European Nucleotide Archive (ENA; PRJEB39599) to reconstruct the phylogeographic history of Holarctic nine-spined stickleback populations [17].

**Fig. 1:**
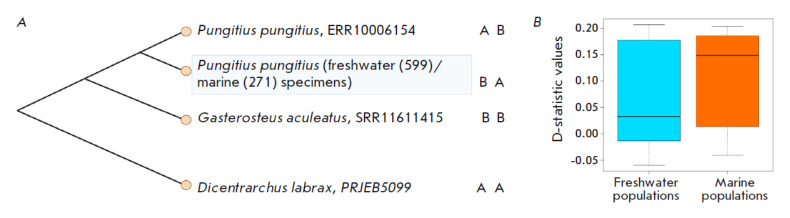
The ABBA-BABA test. (*A*) Schematic interpretation of the four-taxon ABBA-BABA test used to detect introgression between freshwater and marine populations of the nine-spined stickleback and three-spined stickleback. AB – randomly selected freshwater nine-spined stickleback specimens from the dataset obtained by Feng et al. [17]. BA – the marine and freshwater nine-spined stickleback specimens investigated by Feng et al. [17]. BB – the marine three-spined stickleback specimen genomic data obtained earlier by Nedoluzhko et al. [16], AA – European seabass (*Dicentrarchus labrax*), the outgroup. (*B*) D-statistic values distributions among freshwater and marine populations of nine-spined stickleback

We classified the genomic datasets of the ninespined stickleback specimens in accordance with their ecotype into marine (271 specimens) and freshwater (599 specimens) groups. Next, we performed a comparative analysis aiming to assess the level of introgression of the three-spined stickleback into the genotypes of specimens belonging to the marine and freshwater ecotypes of the nine-spined stickleback (*Fig. 1A*).

The introgressive hybridization between the two stickleback species was detected using the ABBA-BABA test [18]; the genome of the European seabass (*Dicentrarchus labrax*, diclab1, PRJEB5099) was used as the outgroup for this test. Initially, the sequencing data for each nine-spined stickleback specimen were mapped to a reference genome of *D. labrax *using the bowtie2 software package (v. 2.3.4.1), with the *very-sensitive-local *parameter [19] (Appendix 1). The mapped data, in SAM format, were then converted to the BAM format, sorted, and indexed using the SAMtools package (v. 0.1.19) [20]. The resulting BAM files were analyzed using the ANGSD software package [21] to evaluate the introgression between the anadromous three-spined stickleback and two ecotypes of the nine-spined stickleback. The statistical significance of the results of the tests for introgression were assessed using the nonparametric Wilcoxon test.

The introgressed loci were revealed using the SNP genotyping chart obtained by mapping the genomic sequencing data to the reference genome of *D. labrax*. A freshwater ERR9997510 specimen, with the minimal D-statistic value, was chosen as the reference genome of *P. pungitius*. The genomic data of the SRR11611426 specimen reported by Nedoluzhko et al. [16] were used as the reference for the genome of *G. aculeatus*. Only the loci homozygous, with respect to the reference allele for the ERR9997510 specimen, and homozygous, with respect to the alternative allele for the SRR11611426 specimen, were analyzed. The alternative allele frequency in all the specimens of the marine ecotype of the nine-spined stickleback was determined in all the filtered loci. In the loci where the alternative allele frequency was > 0.5, the genes were identified by mapping to the reference database of the zebrafish (*Danio rerio*) GRCz11 (https://www.ncbi.nlm.nih. gov/datasets/genome/GCF_000002035.6/), and the blastx software v2.12.0+ [22]. Gene ontology analysis was conducted using the ShinyGO v0.81 web tool [23].

## RESULTS

We have assessed the level of introgressive hybridization of the three-spined stickleback into the genotypes of the nine-spined sticklebacks corresponding to the marine and freshwater genotypes using the ABBA–BABA test. The introgression was evaluated by calculating the D-statistics value for each specimen within each sample (Appendix 1). The mean distribution of the D-statistic values in marine populations of the nine-spined stickleback was 0.1488593, while it was 0.03277605 (near-zero) in the freshwater populations (*Fig. 1B*).

Hence, the level of genomic introgression from the three-spined stickleback to the nine-spined one was significantly higher in the group of marine specimens compared to the group of freshwatetr ones (Appendix 1; *Fig. 1B*). This finding proves the hypothesis that genomic introgression from the three-spined to the nine-spined stickleback, which has largely evolved in fresh water [24] and presumably is not well-adapted to marine water, has an adaptive effect [16].

The nonparametric Wilcoxon test allowed us to assess the statistical significance of the difference in D-statistic values between the marine and freshwater nine-spined stickleback populations (*p-value *= 1.004e-07), revealing a high statistical significance of the differences in the level of introgression in the genomes of marine as compared to freshwater nine-spined stickleback specimens.

The higher level of introgressive hybridization in marine populations can presumably be attributed to the presence of alleles of the anadromous threespined stickleback in their genomes, which have been fixed in marine populations of the nine-spined stickleback, thus facilitating the adaptation of their carriers to higher salinity.

An analysis of the introgressed regions revealed 715 loci where the frequency of the allele specific to the three-spined stickleback is > 0.5 in marine nine-spined stickleback populations. These loci reside in 432 genes. The list of the genes is provided in Appendix 2. Gene ontology (GO) enrichment analysis demonstrated that the list of introgressed genes is enriched in groups of categories related to organism development processes and regulation of transcription, cell adhesion, and transmembrane ion transport (Appendices 3–5). These gene groups, primarily those functionally related to cell adhesion and ion transport, can potentially be associated with salinity adaptation.

## CONCLUSIONS

The progress and cheapening of deep DNA sequencing technologies, as well as the development of fundamentally new bioinformatics analysis methods, make it possible to assess the reasons for the explosive speciation, adaptive radiation, and rapid ecological adaptation, or identify the traces, of ancient genomic hybridization [12, 14, 25].

The fact that introgressive hybridization between the three-spined and nine-spined stickleback in the White Sea basin was possible had previously been demonstrated [15, 16]. Interestingly, distinct signals of introgression from the three-spined stickleback were had been observed in most of the genomes of the studied nine-spined stickleback specimens [16]. However, in the absence of marine nine-spined stickleback specimens, it was impossible to confirm the adaptive potential of such intergeneric introgression. This study has clearly demonstrated, using genomic data on marine and freshwater populations of the nine-spined stickleback and statistical tests, that the marine populations of this species enhance adaptivity to water salinity due to introgression from the three-spined stickleback, a mostly marine species.

Our findings indicate that the available genomic data need reinterpreting from the position that the destruction of reproductive barriers between species, including evolutionarily distant ones, is a much more frequent phenomenon than previously thought. Furthermore, it appears that introgressive hybridization can have a significant adaptive potential during periods of environmental changes, global cataclysms, and mass species extinction [11]. Another conclusion flowing from our results is that genomic introgression events require a more careful consideration as one of the significant factors in evolution. Moreover, introgression should be taken into account when conducting phylogenetic studies and when assessing the demographic history of species, since introgressive hybridization events substantially contribute to them.
